# The relationship between attendance and academic performance of undergraduate medical students during surgical clerkship

**DOI:** 10.1186/s12909-021-02833-2

**Published:** 2021-07-22

**Authors:** Hamdi Al Shenawi, Rami Yaghan, Amer Almarabheh, Noor Al Shenawi

**Affiliations:** 1 Department of SurgeryCollege of Medicine and Medical Sciences, Arabian Gulf University, Manama, Bahrain; 2 Department of Surgery and Urology, Faculty of Medicine, Jordan University of Science and Technology, Irbid, Jordan; 3 Department of Family and Community Medicine, College of Medicine and Medical Sciences, Arabian Gulf University, Manama, Bahrain; 4 Undergraduate medical student, College of Medicine and Medical Sciences, Arabian Gulf University, Manama, Bahrain

**Keywords:** Attendance 1, Achievement 2, Performance 3, Correlation 4, Surgical clerkship 5, Gender 6

## Abstract

**Background:**

The current study aimed to evaluate the previously unexplored correlation between undergraduate medical students’ attendance during their surgical clerkship and their academic performance. It also aimed to explore any difference in the attendance rate between male and female students and whether this difference, if present, affects the academic performance.

**Methods:**

A retrospective descriptive cross-sectional study has been conducted on 331 undergraduate medical students during their surgical clerkships at the College of Medicine and Medical Sciences (CMMS) at Arabian Gulf University (AGU), Bahrain from September 2018 to June 2020.

**Results:**

There was a positive statistically significant correlation between students’ attendance during surgical clerkship and academic performance (r = 0.360, *P* <  0.01). Mean attendance rate was greater in each increasing category of academic performance: 47.95% in the weak category (less than 65%, *n* = 42), 57.62% in the good performance category (65% to less than 75%, *n* = 108), 67.82% in the very good performance category (75% to less than 85%, *n* = 126), 83.16% in the excellent performance category (85% and above, *n* = 55). The mean attendance rate of male students was 59.76% (SD = 25.73), compared to 66.92% (SD = 24.30) in the female students. T-test indicated that the difference between the mean attendance of the two groups of the students (male, female) was statistically significant (t = 2.483, *p* <  0.05). On the other hand, the difference between the mean academic performance for the two groups of students, male & female, (t = 0.284, *p* = 0.777) was not statistically significant.

**Conclusions:**

Our study showed a significant relationship between undergraduate medical students’ attendance during their surgical clerkship and their academic performance. Further studies are needed to stratify this correlation according to clinical and theoretical teaching activities. No significant difference was observed in academic performance between female and male students.

**Supplementary Information:**

The online version contains supplementary material available at 10.1186/s12909-021-02833-2.

## Background

The vast majority of medical literature is indicative of a clear synergistic correlation between medical student attendance, and academic achievement [1–15]. The key for an undergraduate medical student to fulfil academic achievements and rewards is to attend the learning activities and be fully committed. This positive correlation has been observed at all student levels including those with low scoring abilities [1]. Despite this global realization, absenteeism of undergraduate students from medical and health science schools continues to be a major issue negatively affecting student performance [1]. Class participation helps to enhance the student’s understanding of the topics discussed, aids in facilitating class activities, and allows the student to develop ethical and moral values. In addition, attendance is an essential part of providing regulatory perspectives for sound professionalism [2, 16]. Absenteeism entails negative impacts on institutions as well. The university resources are underused, the teaching staff are not motivated, and the student-teacher relationship becomes burdensome [3].

Academic authorities have proposed several causes for academic absenteeism. The most common causes were pop quizzes, long break periods between classes, and the performance of some teachers who resort to just reading the text provided on the slides [3]. Another common cause for absenteeism is having ‘prior- years printed handouts’ prepared by senior students. This theory agrees with some European research that assigned a large percentage of student absences to resources that allow students to access all notes and study material without attending classes [3]. New technologies have aided the increase of absenteeism by allowing more access to information at home. One of the limitations of the arguments for and against obligatory class attendance focuses only on the students’ academic performance and not on the principles and experience a student acquires from the classroom. One study proved that students who attend 40% of the classes practice human values to a higher degree compared to students attending less than 40% of the classes [16].

The above-mentioned correlation between attendance and performance outcome [4], was not specifically studied in undergraduate surgical clerkship programs. Surgical rotations have their own merits. We believe that in medical education, particularly in clinical surgical training, regular attendance of the students enhances the competence and confidence level of the students. In addition, regular attendance helps in increasing student’s exposure to hot and cold surgical cases and having increased opportunities to practice basic surgical procedures. Also, attendance allows the students to understand responsibilities and work dynamics as a member of a surgical team. This study aimed to explore the extent of correlation between attendance and performance among undergraduate medical students during their surgical clerkship in particular. It also aimed to explore any difference in the attendance rate between male and female students and whether this difference, if present, affects the academic performance.

## Methods

### Type of study

A retrospective descriptive cross-sectional study.

### Study target and population

Final year medical students (year six) during their 17-week surgical clerkship for two consecutive full academic years (2018/2019 & 2019/2020). The rotation included general surgery, pediatric surgery, vascular surgery, plastic surgery, urology, orthopedic surgery, anesthesiology, and accident and emergency services.

### Data collection and study instrument

The required data has been collected from the computer database at the department of surgery, and the assessment office at CMMS at AGU, Bahrain. The study period extended from September 2018 to June 2020. 

There were 4 surgical clerkship rotations with a total number of 331 students. Students were expected to attend 124 activities per rotation (44 afternoon problem-based activities, 5 professional skills at Simulation Center, and 75 hospital-based clinical activities).

This 17-week surgical clerkship introduces students to the basic principles of general surgery and surgical subspecialties. It is designed to equip the undergraduate students with the fundamental knowledge, basic skills, and attitudes relevant to a sound surgical practice.

The clinical surgical rotation program has taken place at AGU campus, Salmaniya Medical Complex, and King Hamad University Hospital. These are the largest two governmental teaching hospitals affiliated to CMMS-AGU. Student attendance of all clinical and tutorial-based activities was confirmed using a paper-based logbook. The logbook was checked and signed by the assigned tutor on daily basis. Student attendance was defined as full attendance of the hospital-based activities, simulation center activities, and the problem-based activities at AGU campus. By mid-March 2020, our institution implemented precautionary measurements against COVID-19 dissemination. In this regard, students were not allowed to attend in hospitals. Compensatory teaching activities were immediately introduced using zoom application. These included a variety of small group interactive clinically-oriented activities including case studies, seminars, and video simulations. Attendance of students was assured by making the video-on option compulsory. A dedicated secretary recorded the details of attendance of each student by the continuous monitoring of the screen in each activity. It is worth mentioning here that the training in our institution officially ends by late April during the academic year. May and June will be the examination preparation period, and the examination periods for the clinical clerkships and the general MD graduation exams. This meant that by the time COVID-19 closure has started, the concerned batch of students have already fulfilled two thirds of their regular clinical training.

### Exposures

The attendance rate (as a total percentage) for each student was calculated as a function of the actual number of activities attended to the total number of scheduled activities. All activities were given an equal weight.

The academic performance of each student at the end of each surgical clinical rotation at CMMS-AGU is calculated according to the following percentages: clerkship continuous assessment (25%), short answer questions (10%), multiple choice questions (25%), objective structured clinical examination, (10%) and clinical encounter examination (30%).

The 25% of clerkship continuous assessment has been excluded from calculation because it depends largely on attendance. By this, we have eliminated the potential bias that might affect our statistical evaluation of the correlation between attendance and academic performance. This also reduces the subjectivity of the tutors’ own perception. The remaining 75% of the academic performance has been converted to 100% in our calculation.

### Statistical analysis

The Data has been entered in MS Excel and analyzed using Statistical Package for the Social Sciences (SPSS Version 27). Study Variables: The dependent variable is academic performance (scores), and the independent variables are attendance and gender. Variables have been presented as counts and percentages or as means and standard deviations where applicable. Two independent samples t-tests were used to test the significant mean differences in student performance in surgical clerkship rotation scores regarding the percentage of attendance and gender.

The Pearson correlation coefficient was used to measure the linear relationship between the surgical clerkship rotation score and the percentage of attendance. A scatter diagram and a simple regression line were used to present the association between two previously quantitative variables. Chi-square test was used to measure the association between students’ attendance and student’s performance in clerkship rotation, and gender. Additionally, the chi-square test was used to measure the association between students’ performance in clerkship rotation, and attendance. A *p*-value of less than 0.05 was considered statistically significant.

## Results

A total of (331) final year medical students (Table 1) completed the 17- week surgical rotation during the study period. Out of these, 111 (33.5%) were males, and 220 (66.5%) were females. The mean attendance rate of surgical clerkship rotation ± SD was 64.53% ± 24.98%, and the mean of overall academic performance ± SD was 75.12% ± 11.33%.

**Table 1:** Total number of the targeted students

Gender	Four Surgical Clerkship Rotations – Year 6				
	R1	R2	R3	R4	Total
Male	31	32	23	25	111
Female	53	58	53	56	220
Total	84	90	76	81	331

### Relationship between attendance rates and academic performance

**Fig. 1:**
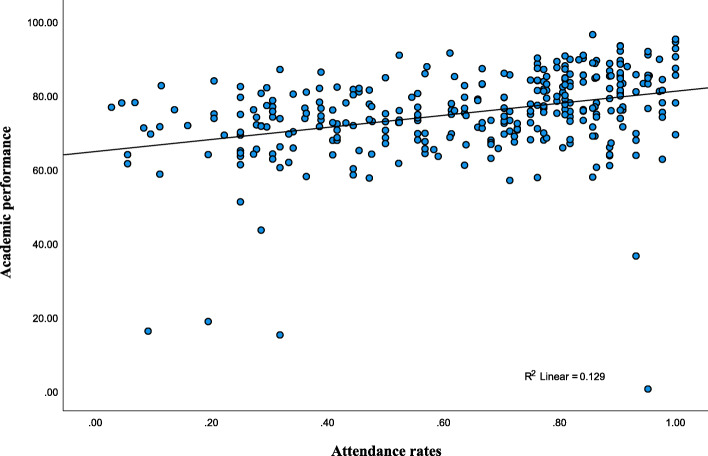
Scatter plot of the positive linear correlation betwwen attendance and performance

The results of Pearson correlation coefficient are shown in (Fig. 1). There was a positive statistically significant correlation between surgical clerkship attendance, and academic performance (r = 0.360, *P* < 0.01).

### Attendance rate according to performance levels

**Fig. 2:**
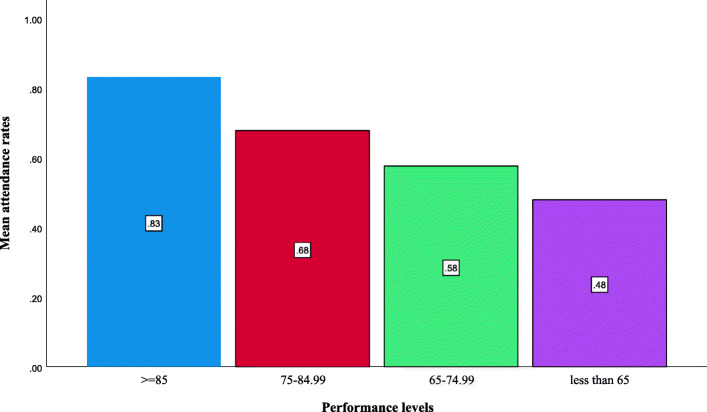
The mean of attendace rate according to the academic performance levels

Mean attendance rate was greater in each increasing category of academic performance: 47.95% in the weak category (less than 65, *n* = 42), 57.62% in the good performance category (65% to less than 75%, *n* = 108), 67.82% in the very good performance category (75% to less than 85%, *n* = 126), and 83.16% in the excellent performance category (85% and above, *n* = 55) (Fig. 2).

### Academic performance according to attendance categories

**Fig. 3:**
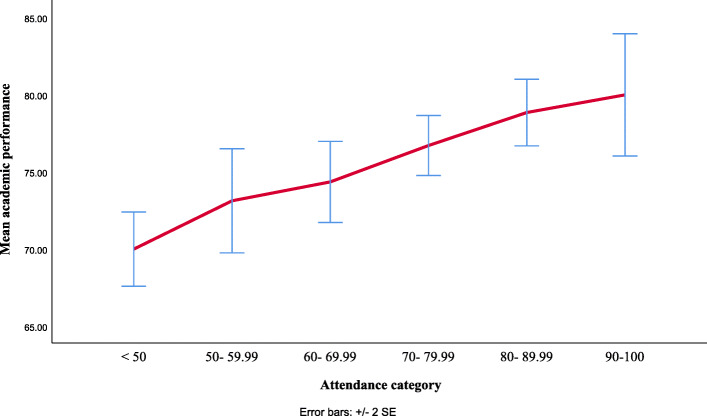
The mean of academic perforamnce score according attendance rate category

The mean student performance was greater in each increasing category of attendance: 69.97% (SD = 12.16%, *n* = 102) in the 50% or lower category, 73.10% (SD = 7.91%, *n* = 22) in the 50 to 59% category, 74.33% (SD =7.32%, *n* = 31) in the 60 to 69% category, 76.69% (SD = 7.27%, *n* = 56) in the 70 to 79% category, 78.83% (SD = 8.71%, *n* = 65) in the 80 to 89% category, and 79.67% (SD = 14.67%, *n* = 55) in the 90 to 100% category (Fig. 3).

### Gender-based attendance rate and academic performance score

The mean attendance rate of male students ± SD was 59.76% ± 25.73%, compared to 66.92% ± 24.30% in the female students, (Table 2). The results of t-test indicated that this difference was statistically significant (t = 2.483, *p* < 0.05). On the other hand, the difference between the mean of academic performance for the two groups (male, female) students (t = 0.284, *p* = 0.777) was not statistically significant.

**Table 2:** Gender-based attendance rates and academic performance score

Variable	Male (***n*** = 111)	Female (***n*** = 220)	T. value	***P*** value
	Mean ± SD	Mean ± SD		
**Attendance rate (%)**	59.76 ± 25.73	66.92 ± 24.30	2.483	0.014*
**Academic Performance**	75.37 ± 12.28	74.99 ± 10.85	0.284	0.777

*: p < 0.05

### Factors associated with attendance rates

According to the attendance rate, students were divided into two categories; those who attended ≤50%, and those who attended > 50%. According to the student’s performance, the students were divided into four categories: excellent (≥ 85), very good (75 to < 85), good (65 to < 75), and weak (< 65). Chi-square tests (Table 3) showed that there was a significant association between attendance category and academic performance level (*p*-value < 0.001). On the other hand, the association between attendance category and gender was not statistically significant (p-value > 0.05).

**Table 3:** The association between academic performance, gender, and attendance rate category

Variable	Performance Level	Attendance ≤ 50%.		Attendance > 50%		Total	P value
		n	%	n	%		
**Academic Performance**^a^	Excellent	9	5.3	46	28.4	55	< 0.001
	Very Good	54	32.0	72	44.4	126	
	Good	74	43.8	34	21.0	108	
	Weak	32	18.9	10	6.2	42	
	Total	169	100	162	100	331	
**Gender**	Male	65	38.5	46	28.4	111	0.052
	Female	104	61.5	116	71.6	220	
	Total	169	100	162	100	331	

^a^: Excellent (≥ 85), Very good (75 to < 85), Good (65 to < 75), Weak (< 65)

Previous performance might be a potential confounding variable associating better performance to an underlying characteristic of the learner, rather the attendance rate. We have compared the scores of the high performing group in surgery in the current study (*n* = 55), to their previous scores in internal medicine during their fifth year using the paired sample t-test. The mean in the surgery clerkship was (88.42, SD = 2.82), compared to (80.92, SD = 6.82) in the internal medicine. The paired sample t-test indicated that this difference was statistically significant. (Mean Diff. = − 7.497, 95% C. I = − 9.205 to − 5.789, *p* < 0.001). Out of the 55 students falling in the high-performance category in the current study, only 32.72% achieved a similar high-performance score-range in internal medicine.

## Discussion

The preponderance of evidence indicates a clear positive correlation between medical student attendance in general, and the learning outcome achievement and student scoring (Table 4). However, there is no previous data regarding such a correlation during the surgical clinical clerkship in particular.

**Table 4:** A summary of representative publications evaluating the relationship between student’s attendance, gender, and academic performance

Author, year of publication	Relationship between attendance & academic performance	The difference in academic performance & attendance according to gender
Current study Al Shenawi et al.	+ (positive correlation)	No significant effect but Females have more attendance than males
Khan YL et al., 2019 [1]	+ (positive correlation)	Not applicable
Thatcher A et al., 2007 [2]	+ (positive correlation)	Not applicable
Landin M et al., 2015 [3]	+ (positive correlation)	Not applicable
Deane RP et al., 2013 [4]	+ (positive correlation)	Not applicable
Al Khaja KA et al., 2019 [5]	+ (weak correlation)	No significant effect
Subramaniam BS et al., 2013 [6]	+ (positive correlation)	Not applicable
Daud S et al., 2012 [7]	+ (positive correlation)	Female students have better attendance rate & academic performance than male
Dhaliwal UP et al., 2003 [8]	+ (positive correlation)	Not applicable
Khan HU et al., 2003 [9]	+ (positive correlation)	Not applicable
BinSaeed AA et al., 2009 [10]	+ (positive correlation)	Not applicable
Sharmin T et al., 2016 [11]	+ (positive correlation)	Not applicable
Sharma S et al., 2019 [12]	+(strong correlation)	Academic performance of female students better than males
Roy SS et al., 2014 [13]	+ (weak correlation)	Academic performance of female students better than males
Nowreen N et al., 2019 [14]	+ (positive correlation)	Not applicable
Silva ET et al., 2010 [15]	+ (positive correlation)	Not applicable
Cheruvalath R et al., 2017 [16]	+ (positive correlation)	Not applicable
Fadelelmoula T et al., 2018 [17]	+ (positive correlation)	No significant effect
Ayodele OD et al., 2017 [18]	+ (positive correlation)	No significant effect
Marburger DR et al., 2006 [19]	+ (positive correlation)	Not applicable
Schnee D et al., 2019 [20]	+ (positive correlation)	Not applicable
Lukkarinen A et al., 2016 [21]	+ (positive correlation)	Not applicable
Aldamen H et al., 2015 [22]	+ (positive correlation)	Not applicable
Kauffman CA et al., 2018 [23]	No correlation	Not applicable
Yeager L et al., 2018 [24]	No correlation	Not applicable
Faisal R et al., 2017 [25]	Not applicable	No significant effect
Albalawi M et al., 2019 [26]	Not applicable	Academic performance of female students better than males
Cortright RN et al., 2011 [27]	Not applicable	Attendance on academic performance is more significant for female students than males.

We believe that the undergraduate surgical clinical training has some inherent properties that differentiate it from other disciplines and intensify the necessity of student physical attendance in the clinical hospital-based and classroom-based learning activities. Most patients with cold surgical problems and specific physical signs would visit the hospital for a short period only once or twice before having their definitive curative surgery. This will naturally reduce the student chances to see surgical cold cases, calling for a more dedicated physical student attendance. The nature of the cold cases in surgery also reduces the chance of creating a comprehensive s*tandardized patient (SP)* program. A patient with a hernia, for example, will be advised to have his hernia repaired as soon as possible. This is different from a patient with mild or moderate aortic incompetence who can be enrolled in a controlled SP program for timely student training.

Also, surgical training includes a wide range of clinical skills (like wound follow-up, simple catheter insertions and removals, and the multidisciplinary operating theatre approach), which can only be monitored by direct face-to-face and, occasionally, supervised hands-on practicing. On the other hand, emergency surgical cases will most of the time be operated upon within a short period, limiting the student’s chances to witness the real initial clinical presentation.

Exposure of medical students to real cold and emergency surgical cases gives them a good chance to learn how to assess these cases properly, make quick logical decisions, build their communication skills, obtain consent from patients, and train on breaking bad news.

The specific surgical training features alluded to above, prompted us to investigate the correlation between attendance and performance during the surgical training as a separate discipline.

Our study revealed a significant positive correlation between undergraduate medical students’ attendance and academic performance during their surgical clerkship (Tables 2,3). These findings are consistent with most of previous reports assessing the correlation between attendance and academic performance but in other disciplines [1–22] (Table 4). However, Kauffman CA et al. and Yeager L et al. found no correlation between attendance and academic performance [23, 24].

We believe that the undergraduate medical students in their clinical surgical rotations should be strictly followed with a close attendance monitoring system for a better outcome. In our institution, a logbook is used by each student and is signed daily by the assigned responsible tutor after the student physically fulfills the requested task. In our institution, the continuous student assessment is also partially dependent on the ratio of attendance. More studies are needed to explore further the specific advantages of strict adherence to attendance during the clinical surgical rotations.

Among our study group, there was no significant difference in academic performance between female and male students during the undergraduate surgical clerkship (Table 2). However, female students had a higher attendance rate compared to male students. Similarly, in this regard, Al Khaja KA et al., Fadelelmoula T et al., Ayodele OD et al., and Faisal R et al. recorded no significant correlation between gender and academic performance in other disciplines [5, 17, 18, 25] (Table 4). However, other studies reported that female students imply a better academic performance compared to male students [7, 12, 13, 26, 27] (Table 4).

Further research is required to have a better understanding of the relationship between gender and academic performance in undergraduate medical students during the clinical surgical clerkship. Previous learner performance is a recognized predictor of future success for learners. If most of our study subjects, falling in the high performing group, had previous high academic performance, then it could equally be stated that high performing students were more likely to have high attendance rates. In other words, previous performance might be a potential confounding variable associating better performance to an underlying characteristic of the learner, rather than merely the attendance rate. To address this more deeply, we have compared. The scores of the high performing group in surgery in the current study, to their previous scores in internal medicine during their fifth year. The mean score in the surgery clerkship was (88.42), compared to (80.92) in the internal medicine clerkship. This difference was statistically significant. (Mean Diff. = − 7.497, 95% C. I = − 9.205 to − 5.789, *p* < 0.001). This favors the conclusion that, within our study group, the attendance rate was the main predictor of better performance compared to previous scores in other disciplines.

### Limitations

Our study included a relatively small number of students calling for further studies.

Among our study group, performance results were not segregated according to clinical hospital-based and theoretical classroom-based activities, creating another issue for future studies.

## Conclusion

In consistency with other medical disciplines, there was a positive correlation between undergraduate student attendance during the surgical clerkship and academic performance. No significant difference was observed in academic performance between female and male students. Further studies are needed to segregate the relationship between student’s attendance, gender, and academic performance according to clinical and theoretical teaching activities.

## Supplementary Information

**Additional file 1.** “Article title, Author, and affiliations”.

**Additional file 2.** “Result of Surgery Exams -R1, R2, R3, R4” Data not shown.

## Data Availability

All data generated or analysed during this study are included in this published article [and its supplementary information file -Results of Surgery Exams R1, R2, R3, R4].

## Abbreviations

CMMS: College of Medicine and Medical Sciences
AGU: Arabian Gulf University
SD: Standard deviation
SP: Standardized patient
R1: surgical rotation 1
R2: surgical rotation 2
R3: surgical rotation 3
R4: surgical rotation 4
