# Supplementary material for: The relationship between attendance and academic performance of undergraduate medical students during surgical clerkship
Source: BMC Med Educ. 2021 Jul 22;21:396. doi: 10.1186/s12909-021-02833-2 (PMC8298040; doi:10.1186/s12909-021-02833-2)
Supplement: Supplementary file 1 — Additional file 1. “Article title, Author, and affiliations”. [file 12909_2021_2833_MOESM1_ESM.docx]

**The relationship between Attendance and Academic Performance of Undergraduate Medical Students During Surgical Clerkship**

**Hamdi Al Shenawi**^1^***, Rami Yaghan**^1,2^**, Amer Almarabheh**^3^ **and Noor Al Shenawi**^4^

1. Department of Surgery, College of Medicine and Medical Sciences, Arabian Gulf University, Bahrain
2. Department of Surgery and Urology, Faculty of Medicine, Jordan University of Science and Technology, Irbid, Jordan
3. Department of Family and Community Medicine, College of Medicine and Medical Sciences, Arabian Gulf University, Bahrain
4. Undergraduate medical student, College of Medicine and Medical Sciences, Arabian Gulf University, Bahrain

***** Correspondence: [hamdims@agu.edu.bh](mailto:hamdims@agu.edu.bh); Tel.: +973 39699150
